# On a New Practice in Acute and Chronic Rheumatism

**Published:** 1831-09

**Authors:** J. K. Mitchell

**Affiliations:** one of the attending Physicians of the Pennsylvania Hospital.


### RHEUMATISM.

*On a New Practice in Acute and Chronic Rheumatism.* By J. K. Mitchell, m.d., one of the attending Physicians of the Pennsylvania Hospital.*

In the autumn of 1827, a patient affected with caries of the spine was suddenly attacked with all the usual symptoms of acute rheumatism of the lower extremities. One ankle, and the knee of the opposite leg, tumefied, red, hot, and painful, afforded as fair a specimen of that disease in its acute stage as is usually met with. The usual treatment by leeches, purgatives, and cooling diaphoretics, with evaporating lotions, had the effect of transferring the symptoms to the other ankle and knee, and finally to the hip. Disappointed in the treatment, I began to suspect that the cause of the irritation might lie in the affected spine. The difficulty of cure, the transfer of irritation from one part of the lower extremities to another, without any sensible diminution of disease, and the fact of the existence of caries in the lumbar vertebræ, which lie near the origin of the nerves of the lower extremities, rendered probable the opinion that in the spinal marrow lay the cause of this apparently indomitable and migratory inflammation. Under this impression, I caused leeches to be applied to the lumbar curve, and followed them by a blister placed on the same spot. Relief promptly followed these remedies, and the pain, ceasing to be felt in the limbs, was perceived only in the immediate vicinity of the spinal curve. After the blistered surface recovered its cuticle, a few leeches placed over the diseased spine removed the pain, and left the patient in the usual state of indifferent health attendant on such forms of spinal disease.

Striking as were the benefits of the applications made to the spine in a case of apparent inflammatory rheumatism, they did not lead my mind at the time to the general conclusions which, viewing the case as I now do, they ought to have suggested.

In the beginning of the ensuing winter, another case of a similar kind presented itself. A little female patient, having curvature of the cervical vertebræ, was attacked in the night with severe pain in the wrist, attended with redness, tumefaction, and heat. As, on the appearance of these symptoms, the pain in the neck, to which she was accustomed, subsided, I easily persuaded myself of the spinal origin of this inflammation, and accordingly applied leeches to the cervical spine,

* American Journal of Med. Sciences.

with the effect of procuring a prompt solution of the disease of the wrist.

This case led me very naturally to the reflection, that perhaps other cases of rheumatism might originate in the medulla spinalis, and depend on an irritation of that important organ. In the following spring, an opportunity of testing by practice the truth of this opinion presented itself.

William Curran had been upwards of two years afflicted with a rheumatism of the lower extremities, which gradually deprived him of the use of his limbs, and finally confined him to his chamber. Regular medical aid, and many empirical remedies, had been procured, without an abatement of the pain, which became at length almost intolerable.

On my first visit, I found him in his room, in a paroxysm of pain. His legs were swelled from knee to ankle, and the enlargement of the periosteum and integuments gave to the anterior face of the tibia an unnatural prominence: in that place the pain and tenderness on pressure were particularly developed. He was also suffering severely from pain in the scalp, which had existed for a short time previously, and was at length almost insupportable. Along with these symptoms appeared the usual febrile action, with its concomitants.

Notwithstanding the significant hints given by the spine cases referred to, I treated this case for a time in the usual manner: depleted freely, purged actively, blistered the head, and, having caused an abatement of fever, administered corrosive sublimate and decoction of sarsaparilla. Defeated in all my efforts, I at length suggested to my patient the possibility that his disease was so unmanageable because we had not applied our remedies to the true seat of disease, and that, by addressing our measures to the spine, success might yet be found. Accordingly, on the 16th of February, 1828, nine days after my first visit, I had him cupped at the back of the neck, and, as he could not bear any more direct depletion, inserted a large seton over the lumbar spinal region. The cupping, followed by blisters to the back of the neck, relieved his head; and, as soon as the seton began to suppurate freely, his legs became more comfortable. From the 25th, nine days after the insertion of the seton, I visited him but seldom, although I had seen him once or twice a day until that period. Indeed, I paid him but seven visits after the 25th: the last was on the 30th of March. Soon afterwards he resumed his usual pursuits, and about the beginning of June the seton was removed. Since that time he has not had a return of his complaint, and is, at the date of this paper, in the full and vigorous exercise of all his physical faculties.

I could scarcely doubt as to the cause of the cure in this case, because the treatment applied to the spine was that alone which had not already been fully and fairly tried, either by me or those who had preceded me. Indeed, the last applications were made with some hope of success, and the grounds of that hope were expressed to the patient, who was fully persuaded that the spinal treatment was the chief, if not the sole, agent of restoration.

No other well-marked case of rheumatism presented itself in my private circle of practice, until, in the winter of 1830, Mr. Teale's work on Neuralgic Diseases reached this country, and began to attract towards the spinal marrow a greater share of medical attention. Although in his essay I found nothing directly calculated to sustain me in the opinion I felt disposed to adopt concerning the spinal origin of rheumatism, I rose from its perusal with increased confidence in that opinion, and resolved to experimentally examine its truth. The first well-marked case of simple inflammatory rheumatism which subsequently presented itself, was the following:

Robert Gordon, fifty-six years of age, of vigorous constitution and active habits, was the subject of the attack. Observing a severe pain in his right heel and ankle, immediately followed by redness, heat, and tumefaction, he caused himself to be largely bled, and took some salts and magnesia. On the following day the pain and swelling increased, and, the ankle and knee of the opposite limb becoming similarly affected, he was confined to bed.

On the third day my first visit was made: the patient had then a full, strong, frequent pulse, flushed face, dry skin, whitened tongue, and complained much of the severity of the pain in his legs, and of his incapacity of enduring the slightest pressure or motion. As he had already been purged and had used a lotion, I directed the application of seventeen cups to the lumbar region, so as to abstract twelve or sixteen ounces of blood.

Next morning, found the pain almost entirely gone; does not complain of moderate pressure, and is able to move his legs without inconvenience. Ordered a draught of salts and magnesia, with an evaporating lotion of camphor in alcohol.

Third day. Pain in legs scarcely perceptible, but the shoulders, elbows, and wrists are beginning to exhibit marks of severe inflammation, expressed by pain, tumefaction, heat, and redness. Ordered twelve cups to the cervical spine.

Fourth day. The patient sits up, complains of stiffness, but no pain except on one wrist, and that very slight. Directed Epsom salts and magnesia.

Fifth day. Finding nothing for which to prescribe, arranged the patient's diet, recommended the occasional use of aperients, and took leave of the case.

Called, on the tenth, to inquire into results, and found that there had been no return of disease.

Since that time a very severe winter has passed, during which the subject of this report has continued in his customary health, and in the pursuit of his usual employments.

The reader will, in the above case, perceive that the general bleeding, though very copious, proved of no service, and that the large local depletion of the lumbar region benefited solely that part of the disease which lay at the peripheral extremities of the nerves supplied by the lower end of the spinal marrow. The inflammation in the upper extremities continued afterwards in progress, and was arrested only when cups were placed over the cervical end of the spinal column.

The whole case exhibits a fine exemplification of the difference in the character and extent of the influence of general and topical depletion, and proves that local bloodletting is most potent, when applied to that part of the spine which supplies with nerves the parts in a state of active inflammation.

As I feel, in common with the profession, a greater confidence in hospital reports, especially when made by those who are not, by interest or reputation, blinded or misled, I shall present the history of some cases treated after the new method, as drawn up at my request, by Dr. Stewardson and Dr. Norris, the resident physicians of the Pennsylvania Hospital.

The following case, reported by Dr. Thomas Stewardson, is peculiarly interesting, because of its evident dependence on irritation of nervous masses, and the immediate and perfect remedial action of the local applications.

Case I. William Anderson, coloured man, a seaman, aged fifty, was admitted into the Pennsylvania Hospital on the 31st of December, for a chronic rheumatism of upwards of five years' duration. Occasionally the disease intermitted, but generally continued to affect him during the cold season. The pain affected, at one or at various times, almost every part of his right side from head to heel, but had in no case at any period crossed to the opposite side. Like other cases of chronic rheumatism, it was most severe in cold weather, and when warm in bed. According to his statement, he seldom suffered from a winter attack for a less period than three or four months, and the existing exacerbation had lasted only a few weeks.

On the 2d of January, two days after his admission, eight cups were applied to the back of the neck and left side of the head, and a powder was taken, consisting of guaiacum and nitrate of potassa, of each ten grains, with directions to repeat it three times a day.

On the 3d, " pain in the head and arm completely gone; leg no better."

On the 4th as on the 3d. A blister to the nape of the neck, and eight cups over the lumbar spine.

On the 5th. " Says the cups almost immediately relieved the pain in his leg. He now feels perfectly well."

On account of the extreme rigour of the season, the patient was not discharged until the latter part of February, during which period be remained entirely free from disease.

Case II. Jane Black, aged sixteen, was admitted into the hospital on the 9th of March, 1831. About four weeks antecedently, she perceived pain, tumefaction, and a sense of numbness in her feet and ankles, which gradually deprived her of locomotion, and on the third or fourth day confined her to bed. On the second day after the attack, her wrists and hands were similarly affected. In the course of a week her wrists, fingers, and ankles became flexed and rigid, feeling pain from every attempt to straighten them. Such was her condition when admitted. She states that she is of a costive habit, and had been amenorrhagic for two or three months before the appearance of rheumatism. The previous treatment consisted, as she said, of a blister to the umbilical region, and some powders and drops. On her admission, Dr. Norris applied six cups to the cervical, and six to the lumbar spine, which " took away entirely the pain."

On the following day Dr. Otto saw her, and recommended a continuance of the treatment, and accordingly four cups were applied to the upper, and four to the lower part of the spine, with the effect of enabling her to extend her wrists, and to grasp, though imperfectly, with her hands.

On the 11th, took Epsom salt.

On the 13th, spine cupped as before, and a dose of magnesia directed. After the cupping today, she begins to observe a " pricking sensation, as if her feet and hands were asleep."

On the 16th, cups as before.

On the 18th, find her free from pain and tumefaction, recovering gradually the use of her hands, experiencing no uneasiness on motion or pressure. She is unable to stand, because her feet " slide from under her;" but the attempt gives no pain. Besides the remedies already mentioned, soap liniment was applied twice a day to her wrists and ankles.

*Remarks*. In this highly interesting case, the complication of rheumatic irritation with numbness and enfeebled condition of the extensors of the hand, and the flexors of the foot, amounting almost to paralysis, emphatically directs us to the centrically nervous origin of this disease.

Case III. "William White, seaman, aged fifty-two, was admitted November 27th, for rheumatism. He stated that he had an attack in the preceding winter, which had confined him to bed for five months, and that the present affection had commenced with equal severity. On admission, his wrists and arms were tumid and painful, and he complained also of pain in the lumbar region and lower extremities. Cups were applied to his spine, and repeated at proper intervals, two or three times, without the use of any auxillary remedies. The relief was almost complete, when, in consequence of some accident, he was affected with fever and pain in the head, for which he was cupped and blistered at the nape of the neck, and a saline purgative given. Being relieved from the cephalic irritation, he began in a few days to complain again of pain in the feet and ankles, which appeared hot and tumid. Cups having been applied to the base of the spine, entire exemption from pain ensued. The severity of the season prevented his discharge until the 26th of February; but for more than a month before, he had ceased to feel any other inconvenience than a very slight soreness on the top of his feet, and that only when walking. That pain left him previous to his discharge."

This case is reported by Dr. Stewardson.

Case IV. "William King, a seaman, was admitted for a surgical disease, for which he used venesection and low diet, followed by balsam of copaiba and cubebs.

"On the 24th, he was seized with severe rheumatic pain in his left side and shoulder. For this he was twice bled largely, and put under the use of sarsaparilla and nitrous powders, and afterwards of Dover's powders. A stimulant liniment was also applied to the affected part. Under this treatment he remained until the 6th of February, when the pain appeared to be fixed in both the side and shoulder, and he had not been benefited in any way by the remedies employed.

"On the 7th of February, all other remedies being discarded, twelve cups were applied to the spine.

"8th. Pain relieved. Cups to be reapplied.

"11th. Patient states that the last cupping has almost entirely removed the pain from his shoulder, but has not benefited that of his side. Ordered eight cups to dorsal spine.

"13th. No change after last cupping. Cups to be again applied.

"16th. The pain in the shoulder left the patient soon after the application of the cups on the 13th, and has not returned.

"As the pain in the side was confined at last to a small surface, and had been constant for some time, a few cups were applied immediately over it, with beneficial effect. The repetition at length entirely removed it."

This case is reported by Dr. G. Norris.

*Remarks.* The practical interest of this case consists in the total failure of the most judiciously selected remedies of the current practice, and the facility with which the disease, so obstinate before, began to yield to the very first application of cups. To those who still maintain the identity of the effect of general and topical depletion, this case presents a striking difficulty.

Case V. "William Brown, seaman, was admitted March 5th, 1831, for rheumatism. Three months ago he was exposed at sea to great hardships in an open boat. On the day after he was picked up, he felt pain in his shoulders and elbows, which remained until after his arrrival in port, and then suddenly attacked his lower extremities, while entire exemption from pain was experienced in his upper ones. On admission, he complained of pain in the whole course of his legs, but finds it particularly severe in his knees and ankles. The right ankle is swollen, hot, and very painful. Directed the application of ten cups to the small of the back.

"March 6th. Is no better. On examination, I found that the cups had not been placed on the part as ordered, but had been extended to the top of the spine. Therefore ordered another cupping to the loins.

"7th. Was relieved by the cups for a time, but the pain has returned. Cups to be repeated.

"8th. Has had very little pain since the last scarification. The tumefaction of the right ankle has disappeared, and the heat and pain have entirely gone from it.

"On 11th and 13th, in consequence of the reappearance of slight symptoms of the disease, cups were ordered. Their application in both instances afforded relief."

Reported by Dr. G. Norris.

*Remarks.* In the case just recited, the attention of the reader is called to the fact that the cups produced no relief whatever when applied over that part of the spine which did not transmit nerves to the seat of inflammation; thus verifying the important doctrine, that the most potent influence is exerted when our depletory remedies are addressed as nearly as possible to the disease-exciting agent.

Case VI. "Thomas Gordon, a man of colour, a seaman, aged thirty-four, was admitted on the 15th of February, for rheumatic fever. The pain is confined chiefly to his limbs, and his pulse, although excited, is not very active. Ordered ten cups to spine.

"17th. No improvement. It is discovered that the cups had not been placed near the spine, but at a considerable distance on each side of it. Ordered ten cups to dorsal spine.

"18th. The pain in his body and arms diminished, but no improvement observable in his lower extremities, in consequence of which eight cups were applied to the lumbar portion of the spine. For a slight cough, some mucilage was ordered. The patient was relieved by the last cupping, and the pain almost entirely left him. For stiffness in his legs, a stimulant liniment was finally directed.

"On the 1st of March, having been previously apparently cured, his disease suddenly returned. As he had, along with other symptoms of fever, a strong and frequent pulse, sixteen ounces of blood were abstracted, and nitrous powders administered; but as, on the following day, no abatement of the pain of the lower extremities appeared, and though the fever was reduced, eight cups were applied to the lumbar spinal region, which entirely relieved him.

"On the 9th of March, he was discharged cured. After the last scarification, he used for stiffness and weakness of his joints a stimulating liniment."

Reported by Dr. Stewardson.

*Remarks.* In this case several facts are worthy of notice, Twice the cups failed to relieve the lower extremities; once because they were not applied to any part of the spine, and once because they were placed on the dorsal region. The very first application to the lumbar region afforded the expected benefit. In the relapse, a large bleeding and nitrous powders sustained a total failure; while a very moderate quantity of blood drawn from the lumbar region by cups produced an immediate and final solution of the disease.

Case VII. "William Richardson, a seaman, was admitted on the 11th of February, for rheumatism. His attack commenced two weeks before, with pain in the dorsal region and occiput, followed by a sense of numbness, with pain in almost every part of his body. On admission his skin felt cold, his pulse was frequent, tongue slightly coated, and his bowels regular.

"12th of February. Twelve cups were applied along the spine.

"13th. Has no pain; slight numbness of the legs; no appetite; slightly vertiginous: directed him an ounce of sulphate of magnesia.

"14th. Nausea, for which ordered effervescing draught. For the numbness, directed soap liniment.

"15th. No improvement: the numbness of his hands being especially disagreeable, a few cups were applied to the nape of the neck.

"17th. Find the patient free from pain and numbness.

"For an enlargement of the spleen this patient remains in the hospital, but has not had any relapse."

Reported by Dr. Stewardson.

*Remarks.* The most remarkable feature in this case is the concomitant numbness, and the greater difficulty of removing that than the pain; a fact which is not unfrequently observed in cases of rheumatism. The vertiginous affection, too, is interesting as significant of the irritation of central nervous masses.

Case VIII. "Rebecca Leshler, affected by rheumatism of two weeks duration, exhibited a swollen arm and shoulder, attended with pain and redness. She could elevate her arm only when firmly grasped by the hand of an assistant, when the motion became comparatively easy.

"In the evening of the 5th of March, ten cups were applied so as to extend from the top of the neck downwards, immediately over the spine. On the following morning, the pain was gone, and on the subsequent day every vestige of redness and swelling disappeared. No other treatment was used."

Reported by Dr. Stewardson.

Although other cases might be cited in confirmation of the views here taken, I have not leisure at this time to digest and arrange them: at no very distant period I hope to be able to bring the subject more fully before the profession. I may observe, in general, that, as far as I now recollect, only two cases of apparent rheumatism have, in my hands, either in private practice or in the Pennsylvania Hospital, resisted the treatment recommended in this paper; and both of them were in reality neuralgia, and exhibited no traces of inflammation. One of them was an affection severely painful, located in the bottom of the heel; the other was gastric and intercostal.

The preference given to local depletion over other local measures, arose from the greater apparent success and promptness of its action, which scarcely left any thing to be desired: but cases will occur in which other measures must be used, and in which, perhaps, all measures will fail. We are warranted, however, in declaring our conviction that few failures will happen in thus treating acute rheumatism, and that success will diminish as, passing through chronic rheumatism, we enter on the ground of neuralgia, a disease which sometimes spontaneously disappears, but is scarcely ever, in this city, cured by merely *medical* means: the art of the surgeon occasionally subdues it, and the physician often allays, but seldom removes it. Being paroxysmal, and often slumbering for weeks or months, it is not unfrequently mastered in appearance, though seldom cured in reality.

